# Testosterone insulin-like effects: an in vitro study on the short-term metabolic effects of testosterone in human skeletal muscle cells

**DOI:** 10.1007/s40618-017-0686-y

**Published:** 2017-05-15

**Authors:** C. Antinozzi, F. Marampon, C. Corinaldesi, E. Vicini, P. Sgrò, G. B. Vannelli, A. Lenzi, C. Crescioli, L. Di Luigi

**Affiliations:** 1 Department of Movement, Human and Health Sciences Section of Health Sciences, Unit of Endocrinology, Università degli Studi di Roma “Foro Italico”, 00135 Rome, Italy; 2 Department of Anatomical, Histological, Forensic and Orthopedic Sciences-Section of Histology and Medical Embryology, Sapienza University of Rome, Rome, Italy; 3 Department of Experimental and Clinical Medicine, University of Florence, 50134 Florence, Italy; 4 Department of Experimental Medicine, Sapienza University of Rome, Rome, Italy

**Keywords:** Testosterone, Metabolism, Insulin, Human skeletal muscle cells

## Abstract

**Purpose:**

Testosterone by promoting different metabolic pathways contributes to short-term homeostasis of skeletal muscle, the largest insulin-sensitive tissue and the primary site for insulin-stimulated glucose utilization. Despite evidences indicate a close relationship between testosterone and glucose metabolism, the molecular mechanisms responsible for a possible testosterone-mediated insulin-like effects on skeletal muscle are still unknown.

**Methods:**

Here we used undifferentiated proliferating or differentiated human fetal skeletal muscle cells (Hfsmc) to investigate the short-term effects of testosterone on the insulin-mediated biomolecular metabolic machinery. GLUT4 cell expression, localization and the phosphorylation/activation of AKT, ERK, mTOR and GSK3β insulin-related pathways at different time points after treatment with testosterone were analyzed.

**Results:**

Independently from cells differentiation status, testosterone, with an insulin-like effect, induced Glut4-mRNA expression, GLUT4 protein translocation to the cytoplasmic membrane, while no effect was observed on GLUT4 protein expression levels. Furthermore, testosterone treatment modulated the insulin-dependent signal transduction pathways inducing a rapid and persistent activation of AKT, ERK and mTOR, and a transient inhibition of GSK3β. T-related effects were shown to be androgen receptor dependent.

**Conclusion:**

All together our data indicate that testosterone through the activation of non-genomic pathways, participates in skeletal muscle glucose metabolism by inducing insulin-related effects.

## Introduction

Endogenous androgens [e.g., testosterone (T), dihydrotestosterone (DHT)] influence both reproductive and non-reproductive functions in humans through the regulation of early non-genomic and late-genomic biochemical pathways [1–3]. Whether androgens-mediated long-term effects have been largely characterized, few data exist on short-term effects. We know that acute physical exercise induces a rapid increase in the human serum endogenous levels of several circulating factors such as T [4–6], but the related short-term effects on T-targeted cells, such as skeletal muscle cells, have been not yet characterized. Skeletal muscle is the major tissue involved in glucose metabolism accounting for ≈85% of whole-body insulin-stimulated glucose uptake [7]. Data from animal models show that T influences glucose through non-genomic insulin-like mechanisms in adipocytes, cardiomyocytes and myoblast cells [8–11]. T insulin-like effects on human skeletal muscle cells has been not yet fully investigated. Considering the possible role of T in promoting skeletal muscle cells homeostasis and recovery, particularly during prolonged physical exercise and/or after ending exercise [12], we have hypothesized that the observed acute rapid T increases induced by physical activity could modulate insulin-related early non-genomic pathways, thus impacting on the glucose metabolism and promoting muscle cells metabolic homeostasis and/or recovery. We have investigated the short-term effects of T on human muscle metabolic functions in vitro using human cultures of fetal skeletal muscle cells (Hfsmc) [13] treated with T (100 nM) or insulin (I) (100 nM). The gene expression of glucose transporter type 4, 3 and 1 (Glut4, Glut3 and Glut1, respectively), the translocation of GLUT4 and the activation of I-responsive protein kinase B (PKB/AKT), extracellular-signal-regulated kinases (ERK1/2), mammalian target of rapamycin (mTOR), and glycogen synthase kinase 3β (GSK3β) were investigated.

## Materials and methods

### Chemicals

DMEM/Ham’s F-12 medium (1:1) with and without phenol red, phosphate buffered saline (PBS) Ca^2+^/Mg^2+^-free, bovine serum albumin (BSA) fraction V, glutamine, antibiotics, collagenase type IV, NaOH, Bradford reagent, 4′,6-Diamidino-2-phenylindole (DAPI) and all reagents for western blot were from Sigma-Aldrich (St. Louis, MO, USA). Fetal calf serum was from HyClone (Logan, UT). Testosterone (T6147) and bicalutamide (B9061) were purchased from Sigma-Aldrich (St. Louis, MO, USA). 2-Mercaptoethanol was purchased from Life Technologies, Inc. Laboratories (Grand Island, NY). Antibodies (Abs) for western blot and immunocytochemical analysis: rabbit polyclonal primary anti-phospho Ser2448 mTOR (p-mTOR), mTOR, Ser-473 AKT (p-AKT), AKT, Thr-44, and Tyr-42 ERK (p-ERK1/2), ERK2, Ser21/9 GSK3β, GSK3β, mouse monoclonal primary anti-β actin were from Santa Cruz (Santa Cruz, CA, USA); rabbit polyclonal primary anti-GLUT4. Cy3-labeled secondary antibody were from Jackson Laboratory (Maine, USA), peroxidase secondary Abs, all reagents for SDS-PAGE were from Millipore (Billerica, MA, USA). For RNA extraction TRIzol RNA Isolation reagent was purchased by Ambion™; for reverse transcription 10 mM dNTP Mix, Random Primers, RNaseOUT™ Ribonuclease inhibitor and SuperScript^®^ III Reverse were purchased from Invitrogen. SYBR^®^ Green PCR Master Mix for qPCR was purchased from Life Technologies™ (Applied Biosystems^®^). Primers were from invitrogen. Primers for Glut1: Fw CAAGACTGGAGTCATCATCAATGC, Rev: AGGATGCTCTCCCCAAGC; for Glut3: Fw CTCCTTTTCCGTCGGACTCT; for Glut4: Fw CACCCTCACCACCCTCTG, Rev CTTTTCCTTCCAAGCCACTG; for β actin: Fw CTGAACCCCAAGGCCAAC, Rev AGCCTGGATAGCAACGTACA. Plasticware for cell cultures and disposable filtration units for growth media preparation were purchased from Corning (Milan, Italy).

### Cell cultures

Hfsmc were obtained as previously described [13, 14]. Cells were isolated from 11 fetal skeletal male muscles (four upper and seven lower limbs) obtained after voluntary abortion (10–12 weeks of gestation to set up fetal tissue sample collection previously used [15]). Legal abortions were performed in authorized hospitals and written consent was given by the patients for their human fetal tissue to be stored and used for research. The use of human fetal tissue for research purposes was approved by the Committee for investigation in humans of the Azienda Ospedaliero-Universitaria Careggi, Florence, Italy (protocol no 6783-04). All fetal tissue samples have been handled in the same way and maintained in ice-cold PBS until processed for culture preparation as described elsewhere [13, 14]. Confluent cell cultures were split into a 1:2–1:4 ratio using EDTA-trypsin solution (0.2–0.5%), and used from 5th to 12th passage (5p–12p).

### RNA extraction, reverse transcription and real-time quantitative PCR

For mRNA analysis 35 × 10^3^ Hfsmc were seeded and maintained for 24 h or 5 days in serum-free medium to differentiate in myotubes. Cells before and after differentiation were stimulated with T (100 nM) and I (100 nM) for 24 h in serum-free medium containing 0.1% BSA. Cells in serum-free medium with 0.1% BSA and vehicle were used as control. Total RNA and reverse-transcribed-RNA were prepared as described elsewhere [16]. Single-stranded cDNA was obtained by reverse transcription of 1 µg of total RNA. RT-qPCR was performed using 7500 Real Time System (Applied Biosystems^®^) with SYBR-green fluorophore; 40 ng of cDNA were used as template and cycling parameters were 95 °C for 10 min, followed by 40 cycles of 15 s at 95 °C, 1 min at 60 °C, 30 s at 95 °C, 15 s at 60 °C. Fluorescence intensities were analyzed using the manufacturer’s software (7500 Software v2.05) and relative amounts were obtained using the 2^−∆∆Ct^ method and normalized for the ß-actin. Data are expressed as fold increase vs. control taken as 1.

### Protein expression analysis

For protein analysis, Hfsmc, seeded and maintained in the same conditions as previously reported [17], were stimulated for 15 and 30 min, 2, 6 and 12 h in presence or absence of T (100 nM) in serum-free medium containing 0.1% BSA; cells were treated for 15 min with I (100 nM) for comparison. Androgen receptor antagonism was obtained by pre-treating Hfsmc with 100 nM of bicalutamide for 1 h before T treatments. Cells in serum-free medium with 0.1% BSA and vehicle were used as control. Protein concentration measurement was performed with Bradford Reagent. Protein aliquots (20 μg) were processed, loaded onto 10% SDS-PAGE and transferred on PVDF membranes, according to the procedure previously described [18, 19]. Thereafter, membranes were incubated with primary Abs appropriately diluted in Tween Tris-buffered saline (TTBS) (for anti-p-AKT, anti-AKT, anti-p-mTOR, anti-mTOR, anti-p-ERK1/2, anti ERK2, anti-p-GSK3β, anti-GSK3β 1:1000; for anti-β actin 1:5000), followed by peroxidase-conjugated secondary IgG (1:10,000). Proteins were revealed by the enhanced chemiluminescence system (ECL plus; Millipore). Image acquisition was performed with Image Quant Las 4000 software (GE Healthcare) and densitometric analysis with Quantity One^®^ software (Bio-Rad laboratories Inc.). Western blot analysis was performed in three/four independent experiments with different cell preparations.

### Immunofluorescence microscopy

10^4^ cells were seeded onto glass coverslips in growth medium and maintained for 24 h or 5 days in serum-free medium for differentiation. For GLUT4 localization cells were stimulated for 30 min with T 100 nM and I 100 nM in serum-free medium containing 0.1% BSA. Androgen receptor antagonism was obtained by pre-treating Hfsmc with 100 nM of bicalutamide for 1 h before T treatments. Cells in serum-free medium with 0.1% BSA and vehicle were used as controls. For method specificity, slides lacking the primary Abs were processed. To evaluate GLUT4 translocation cells were fixed with 4% PFA to leave intact plasmalemma and incubated with blocking buffer containing 1% BSA for 30 min at room temperature. Ab GLUT4 (1:100) was incubated overnight, followed by Cy3 conjugated secondary Ab (1.400). DAPI nucleic acid stain was used to dye nuclei (1:10,000). Images were acquired at 60× magnification and slides were examined with Zeiss Z1 microscope and Leica TCS SP2 (Leica, Milano, Italy). For quantification: the percentage of GLUT4 positive cells was calculated by counting the number of stained cells over the total in at least 10 separate fields per slide. Experiments were performed three times with different cell preparations.

### Statistical analysis

The statistical analysis was performed using GraphPad Prism 5 software (GraphPad Software, Inc., La Jolla, CA, USA). *T* test was applied and *P* value <0.05 was considered significant and corrected for comparison using Bonferroni’s post hoc test. Data were expressed as the mean ± standard error (SEM).

## Results

### Testosterone-induced Glut4 mRNA expression in undifferentiated and differentiated Hfsmc

**Fig. 1:**
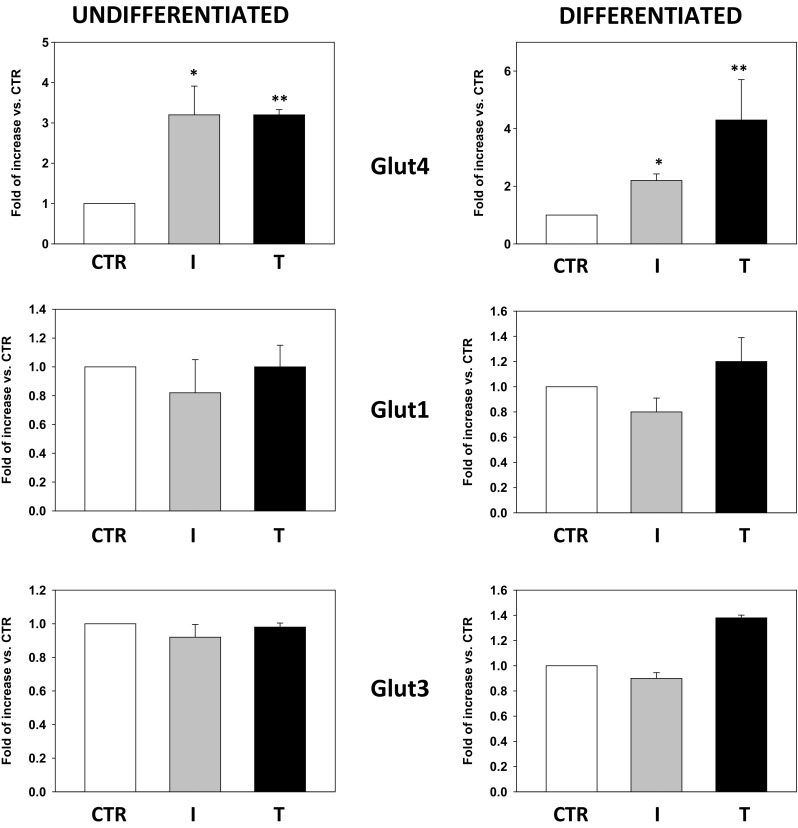
Effect of testosterone on Glut4 mRNA expression. Glut1, Glut3 and Glut4 mRNA expression was evaluated in undifferentiated or differentiated Hfsmc treated for 24 h with T (100 nM) or I (100 nM) for comparison. I or T treatment significantly increased Glut4 mRNA vs. control (c) (*P < 0.05; **P < 0.01); no difference was observed in Glut subtype 1 and 3 mRNA expression. Results (mean ± SE) are derived from three experiments with different cell preparations and expressed as fold increase vs. ctr, taken as 1

mRNA specific for glucose transporter subtypes Glut4, Glut3 and Glut1 was analyzed by qPCR analysis in undifferentiated or differentiated Hfsmc cells, treated for 24 h with T or I, as shown in Fig. 1. T significantly (*P* < 0.01) increased Glut4 mRNA expression in undifferentiated (3.21 ± 0.78 fold of induction vs. c) or differentiated (4.58 ± 0.82 fold of induction vs. c) Hfsmc cells vs. basal expression (control) taken as 1; no changes were observed after I or T in Glut1 and Glut3 mRNA expression vs. control cells. Furthermore, T seems to retain a stronger efficiency in inducing mRNA Glut4 expression vs. I treatment (2.12 ± 0.11-fold of induction vs. control) in Hfsmc differentiated cells.

### Testosterone-induced GLUT4 membrane recruitment in undifferentiated and differentiated Hfsmc

**Fig. 2:**
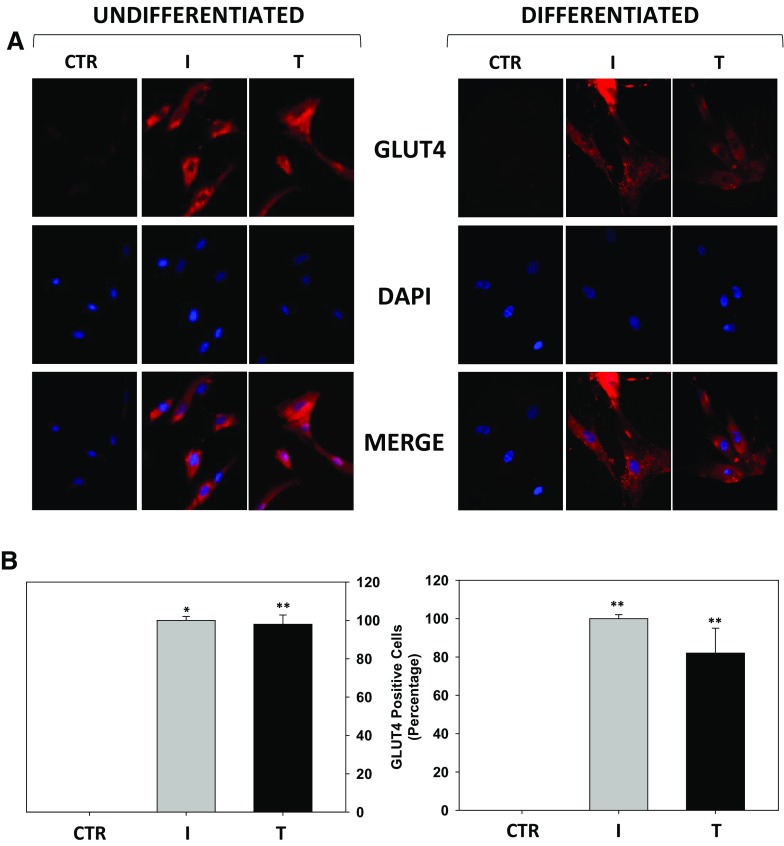
Effect of testosterone on GLUT4 translocation in Hfsmc, before and after differentiation. Immunofluorescence analysis (**a**) revealed no signal for GLUT4 membrane expression in control (ctr) untreated undifferentiated or differentiated Hfsmc; positive staining for GLUT4 was observed after 30-min incubation with I (100 nM) or T (100 nM) both in undifferentiated or differentiated conditions (*upper panels*). Cells were incubated with antibody probes specific for GLUT4, followed by incubations with fluorescent secondary antibody. *Middle panels* represent DAPI blue staining of nuclei; *lower panels* depict GLUT4/DAPI staining merge. Pictures are representative. Results are derived from four separate experiments, using distinct cell preparations. Percentage of GLUT4 positive undifferentiated or differentiated cells (**P < 0.01 vs. ctr) are represented in panels of figure **b**, respectively. Data are expressed as mean ± SE

The effect of T on GLUT4 localization was investigated. Immunofluorescence for GLUT4 performed onto non-permeabilized undifferentiated Hfsmc treated with T for 30 min revealed a stronger positive signal vs. control cells, similarly to I, used as positive control (Fig. 2a, upper panels, undifferentiated).

Similar results were observed in non-permeabilized differentiated Hfsmc (differentiated), where positive specific staining for GLUT4 was stronger after T or I vs. control (Fig. 2a, upper panels, differentiated). Middle and lower panels of Fig. 2a depict, respectively, nuclei DAPI labeling and GLUT4/DAPI fluorescence merging for each conditions.

Percentage analysis of GLUT4 positive cells shows that T-mediated GLUT4 translocation was induced by 99.3% ± 3.1 and 83.4 ± 9.9% in undifferentiated and in differentiated cells, respectively; *P* < 0.01 vs. control. I induced GLUT4 membrane recruitment in virtually 100% of undifferentiated and differentiated cells (Fig. 2b).

### Testosterone rapid effect on insulin-related pathway activation

**Fig. 3:**
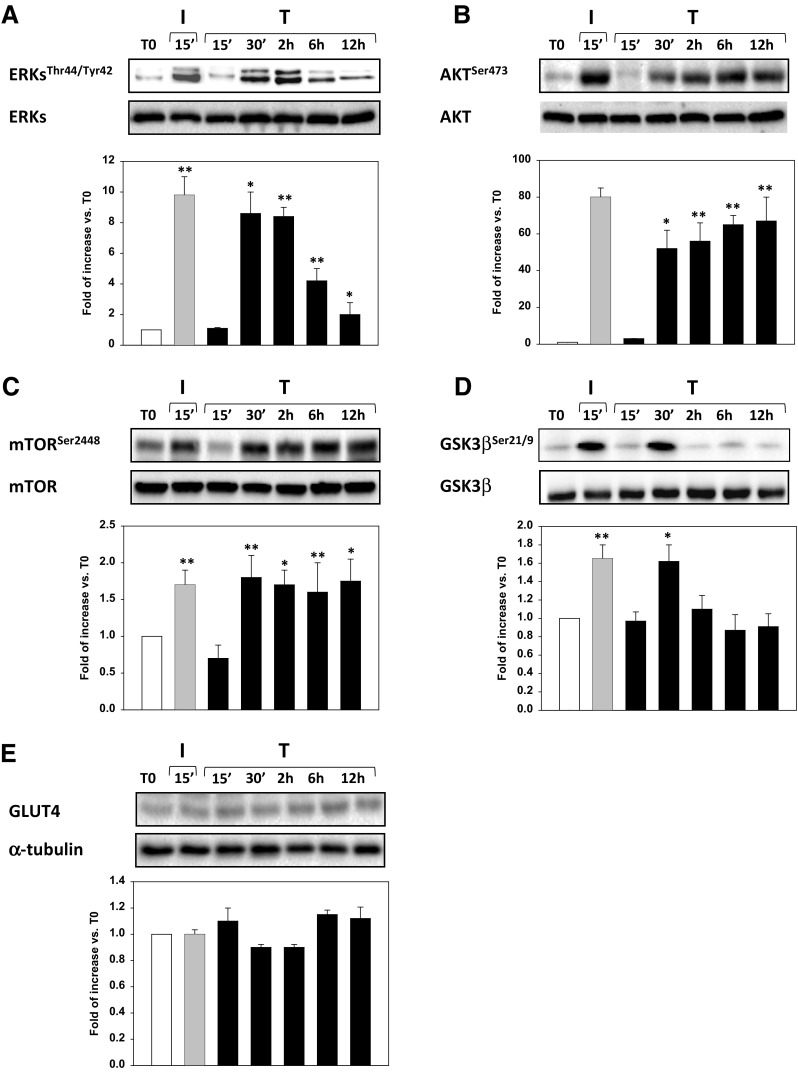
Effect of testosterone on I-related metabolic pathways. The treatment of Hfsmc for 15 min with T (100 nM) did not activate any of the tested intracellular paths, at variance with I (100 nM), used as positive control, (**a**–**d**); however, T after 30 min significantly induced activation of phospho (p)-AKT, ERK1/2, mTOR and GSK3β (*P < 0.05 or **P < 0.01 vs. T0), as depicted by the densitometric analysis, reported below each representative blot. Specific total proteins were used as loading controls. Results of densitometric analysis (mean ± SE) are expressed as ratio p-/total-protein, fold increase vs. T0, taken as 1; data derived from three/four experiments using different cell preparations. Protein analysis revealed no time-dependent changes of GLUT4 expression (**e**)

The effects of T onto I-related signal transduction pathways were investigated in undifferentiated Hfsmc cells treated with T for 15 min, 30 min, 2, 6 and 12 h vs. T0 (time before stimuli addition); 15 min I treatment was used as positive control. T significantly induced a rapid and persistent (30 min to 12 h) phosphorylation/activation of AKT, ERK1/2 and mTOR signaling transduction pathways (*P* < 0.05 or *P* < 0.01 vs. T0) while a transient (30 min) phosphorylation/inactivation of GSK3β (*P* < 0.05 vs. T0) (Fig. 3a–d). No effects were described on GLUT4 protein expression levels (Fig. 3e).

### Bicalutamide counteracts testosterone effects on GLUT4 trafficking and insulin-related pathway activation

The role of AR on T-related GLUT4 trafficking and signaling was investigated in 1 h pre-treated Hfsmc cells with the AR antagonist bicalutamide (Bic, 100 nM) then treated with T (100 nM) for 30; 15 min I treatment was used as positive control.

**Fig. 4:**
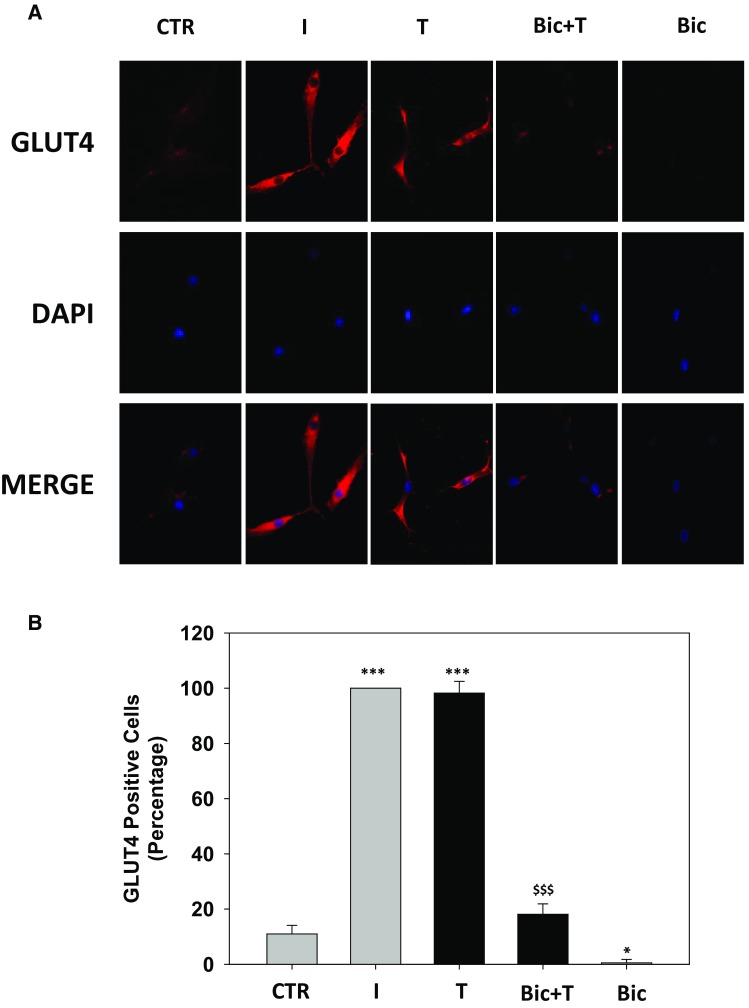
Effect of bicalutamide pre-treatment on testosterone-induced GLUT4 translocation in Hfsmc. Immunofluorescence analysis (**a**) revealed no signal for GLUT4 membrane expression in control (ctr) untreated Hfsmc; positive staining for GLUT4 was observed after 30-min incubation with I (100 nM) or T (100 nM) (*upper panels*). 1-h pre-treatment with Bic 100 nM completely counteracted T-induced GLUT4 translocation. Cells were incubated with antibody probes specific for GLUT4, followed by incubations with fluorescent secondary antibody. Middle panels represent DAPI blue staining of nuclei; *lower panels* depict GLUT4/DAPI staining merge. Pictures are representative. Results are derived from four separate experiments, using distinct cell preparations. Percentage of GLUT4 positive cells (*P < 0.05, ***P < 0.001 vs. ctr and ^$$$^ *P* < 0.001 vs. T) are represented in **b**. Data are expressed as mean ± SE

Immunofluorescence for GLUT4 revealed that Bic pre-treatment counteracted the GLUT4 signal immunodecoration induced by T (Fig. 4a, upper panels). Furthermore, AR antagonism by Bic reduced GLUT4 signal basal levels (Fig. 4a, upper panels). Middle and lower panels (Fig. 4a) depict, respectively, nuclei DAPI labeling and GLUT4/DAPI fluorescence merging for each condition. Percentage analysis of GLUT4 positive cells shows that Bic pre-treatment significantly counteracted T-mediated GLUT4 translocation by 81.82% ± 2.4 (Fig. 4b, *P* < 0.001 Bic + T vs. T).

**Fig. 5:**
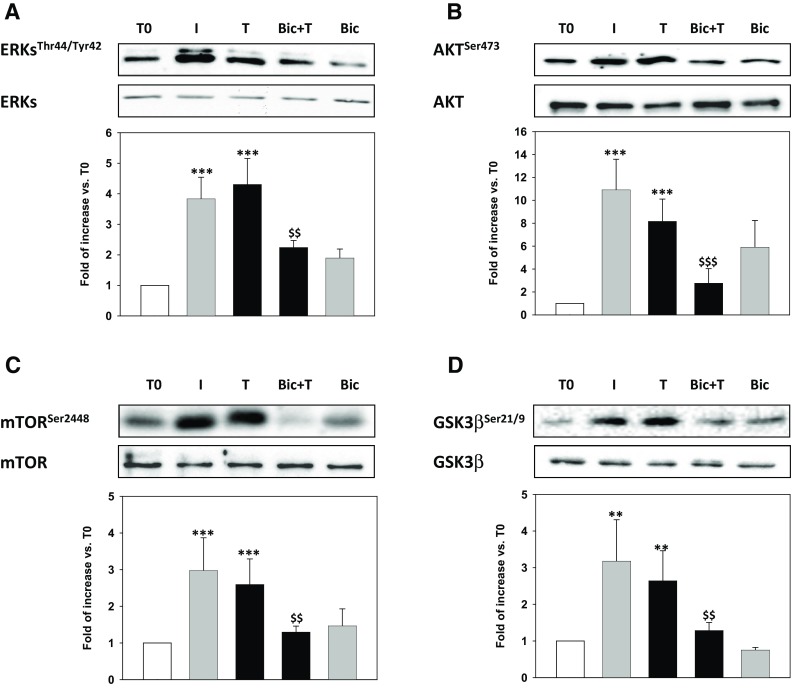
Effect of bicalutamide on Testosterone-induced I-related metabolic pathways. The pre-treatment with bicalutamide (100 nM, Bic) prevented T-induced phosphorylation (p) of AKT, ERK1/2, mTOR and GSK3β (*P < 0.05, **P < 0.01 or ***P < 0.001 vs. T0), as depicted by the densitometric analysis, reported below each representative blot. Specific total proteins were used as loading controls. Results of densitometric analysis (mean ± SE) are expressed as ratio p-/total-protein, fold increase vs. T0, taken as 1; data derived from three/four experiments using different cell preparations

Western blot (Fig. 5) showed that Bic pre-treatment significantly counteracted T-related phosphorylation/activation of AKT, ERK1/2, mTOR and GSK3β signaling transduction pathways (*P* < 0.05 or *P* < 0.001 vs. T) (Fig. 5a–d).

## Discussion

Herein for the first time we showed that T exerts an I-like effect in human skeletal muscle cells, promoting GLUT4 translocation and activating I-dependent signal transduction pathway.

Skeletal muscle system, essential for the postural retention and locomotion has been shown to be an active organ able to dynamically respond to several molecules involved in the regulation of body metabolism, physiologic or pathologic processes, such as T and I, among other factors [5–7, 20, 21]. I, targeting muscle, govern carbohydrate, lipid and protein metabolism [22], while T seemed to mainly affect the body composition [23]. Despite T and I have distinct functions, several evidences suggest their potential interplay both in physiological and in pathological conditions. T-increased levels by physical activity have been shown to play a key role in skeletal muscle tissue homeostasis, metabolism and recovery from exercise-induced stress [9–11, 23]. T lower levels in men affected by hypogonadism and higher in women with polycystic ovary syndrome have been related with an increased risk of metabolic syndrome and diabetes [24–27]. Inversely, type 2 diabetes is frequently associated to reduced T levels [28]. In line with our in vitro results, T has been shown in humans to exert beneficial effects on non-alcoholic fatty liver disease-related insulin resistance, indicating a direct insulin-like effect of testosterone in skeletal muscle cells [29].

Thus, T, for many years considered the “male hormone” with a “muscular hypertrophying function” seems to influence glucose metabolism independently by gender. Starting from these evidences, we documented for the first time in human skeletal muscle cells that T, similarly to I, shortly activates the intracellular machinery committed to metabolic glucose control.

GLUT4 mediates glucose uptake in adipose tissues and striated muscle. After binding to receptor, I induces the rapid (minutes) translocation of GLUT4 to the cell surface that promotes glucose uptake [30] and induces later (hours) effects on Glut4 mRNA expression [31, 32]. Recently, it has been shown that high-dose testosterone promotes GLUT4 translocation and dependent glucose uptake in 3T3-L1 adipocytes cells [9] while no data have been yet collected on human muscle cells. Herein, our results show that in non-permeabilized human skeletal muscle cells, independently from the differentiation status, 30 min of T treatment induced an intense signal specific for GLUT4 protein, virtually absent in control non-treated cells, when GLUT4 is reported to be masked within storage compartments [33], comparable to which described after I treatment. Furthermore, we show that 24 h of T treatment up-regulate Glut4 mRNA expression without affecting Glut1 and Glut3 mRNA levels. To date, 14 glucose transporters isoforms with a specific pattern of tissue expression have been identified [34]. Whether GLUT4 has been found specifically expressed in adipose tissues and striated muscle (skeletal muscle and cardiac muscle), GLUT1 and GLUT3 result, respectively, expressed in erythrocytes [35] or endothelial cells of barrier tissues [36] and in neurons or in the placenta [37]. Our results document the ability of T to regulate, as I, the expression and trafficking of GLUT4 isoform implied in skeletal muscle glucose metabolism.

Following binding to receptor, I activates several signal transduction pathways essential for the GLUT4 recruitment and the regulation of several cell functions, pertinent to metabolic or proliferative effects [38]. Principally, I activates the p21ras/MAP kinase (MAPK) (RAS)/extracellular-signal-regulated kinase (ERK) and the Phosphoinositide 3-kinase (PI3 K)/AKT pathways known to play different role in I-mediated effects. Particularly, whether MAPK/ERK signaling controls cellular proliferation or differentiation, PI3K/AKT influences GLUT4 trafficking and glucose uptake through the phosphorylation-activation of mTOR and -inhibition of GSK3β [39–42]. By time-course experiments, we showed that T induced an early and persistent phosphorylation/activation of PI3K/AKT/mTOR and RAS/ERK paths that starts from 30 min, 15 min later than I, and lasted up to 12 h as well as a transient phosphorylation/inhibition of GSK3β. Notably, PI3K/AKT signaling is also involved in the regulation of GLUT4 protein expression by increased biosynthesis, decreased degradation or both [41]. Herein, T or I treatment increased Glut4 mRNA, but not protein expression. Whether many studies correlate I levels, skeletal muscle cells responsiveness and Glut4 mRNA levels [42], no evidences have still been collected on protein expression status. Recently Ma et al. showed that the prolonged I stimulation down-regulates GLUT4 in 3T3-L1 adipocytes [43]. Analyzing our data we can suppose that T as I increases the GLUT4 protein turnover that is rapidly counteracted by an increase in Glut4 gene transcription and translation. Thus, we speculate that whether it is possible to check up-regulation in mRNA expression no modifications can be described on protein expression levels. The molecular mechanisms by which I and T acutely and chronically regulate GLUT4 half-life will be object of future investigations. All together, the effects herein shown indicate that T is able to induce rapid I-like effects in muscles.

The classical mechanism of T action provides that T binding the androgen receptors (AR) activates AR increasing its affinity for specific DNA-binding sites thus promoting, through the recruitment of co-activators or co-repressors, gene expression regulation. In addition to the classical mechanism, previous studies have determined that androgens can activate cellular signaling pathways independent of AR binding to DNA [1–3]. This “non-classical” mechanism has been shown to increase the phosphorylation/activation of the PI3K/AKT and RAS/ERK pathways in mouse skeletal and rat cardiac muscle fibers [1–3, 43, 44]. Analyzing our data, we can speculate that in human skeletal muscle cells T affects glucose metabolism through the AR-mediated activation of the classic pathway, responsible for Glut4 mRNA expression, and the non-classic pathway that activate PI3K/AKT rapidly predispose cells to glucose uptake. Whether mRNA up-regulation mirrors this rapid effect, belongs to the classic pathway or involves both the ways, has to be elucidated.

In conclusion, our in vitro data provide some biomolecular evidences for I-like effects of T in human skeletal muscle cells, thus sustaining also the role of this hormone in exerting a short-term direct metabolic control on muscle. Considering the observed rapid physiological increases of T during acute physical exercise, the possible presence of both static and dynamic roles of T in supporting metabolic muscle homeostasis and function in resting conditions, during exercise and recovery should be considered. In this sense, we can speculate that in in vivo the T increases rapidly during physical exercise, also throughout its I-like rapid and prolonged effects, that are essential to control skeletal muscle cell function and metabolic homeostasis, particularly during prolonged exercise and/or during recovery. Consequently, further studies are necessary to investigate if these different I-like effects of T are also confirmed in vivo and to verify if during/after exercise they are related to the reached maximum T concentration per se (i.e., does it exist a critical T concentration?) and/or to the observed rapid serum T concentration variation. Besides both the individual responsiveness to T and the role of genetic factors in the physiologic hormones adaptation to exercise-related stress [45], the possible relationships in vivo between T rapid increases and I-like metabolic pathways should be evaluated also in healthy athletes assuming supplements [46] and/or abusing with prohibited [47, 48] and not prohibited [49–52] drugs influencing endocrine-metabolic pathways. Moreover, if our data are confirmed we could also state that athletes often acutely abuse with T also because of the rapid effects on T metabolic I-related pathways, in addition to possible rapid effects of T on neuromuscular system [53]. In fact, from anecdotal reports, athletes could have a benefit for performance by abusing with a single dose of T immediately before or during a competition [54].
